# Rpgrip1 is required for rod outer segment development and ciliary protein trafficking in zebrafish

**DOI:** 10.1038/s41598-017-12838-x

**Published:** 2017-12-04

**Authors:** Rakesh K. Raghupathy, Xun Zhang, Fei Liu, Reem H. Alhasani, Lincoln Biswas, Saeed Akhtar, Luyuan Pan, Cecilia B. Moens, Wenchang Li, Mugen Liu, Breandan N. Kennedy, Xinhua Shu

**Affiliations:** 1 Department of Life Sciences, Glasgow Caledonian University, Glasgow, G4 0BA UK; 2 Key Laboratory of Molecular Biophysics of Ministry of Education, College of Life Science and Technology, Center for Human Genome Research, Huazhong University of Science and Technology, Wuhan, Hubei 430074 P.R. China; 3 Cornea Research Chair, Department of Optometry, King Saud University, PO Box 10219, Riyadh, 11433 Saudi Arabia; 4 Division of Basic Science, Fred Hutchinson Cancer Research Center, Seattle, WA 98109-1024 USA; 5 School of Psychology and Neuroscience, University of St Andrews, St Andrews, KY16 9AJ UK; 6 UCD Conway Institute & UCD School of Biomolecular and Biomedical Sciences, University College Dublin, Dublin, D04 V1W8 Ireland

## Abstract

Mutations in the RPGR-interacting protein 1 (*RPGRIP1*) gene cause recessive Leber congenital amaurosis (LCA), juvenile retinitis pigmentosa (RP) and cone-rod dystrophy. RPGRIP1 interacts with other retinal disease-causing proteins and has been proposed to have a role in ciliary protein transport; however, its function remains elusive. Here, we describe a new zebrafish model carrying a nonsense mutation in the *rpgrip1* gene. *Rpgrip1*homozygous mutants do not form rod outer segments and display mislocalization of rhodopsin, suggesting a role for RPGRIP1 in rhodopsin-bearing vesicle trafficking. Furthermore, Rab8, the key regulator of rhodopsin ciliary trafficking, was mislocalized in photoreceptor cells of *rpgrip1* mutants. The degeneration of rod cells is early onset, followed by the death of cone cells. These phenotypes are similar to that observed in LCA and juvenile RP patients. Our data indicate RPGRIP1 is necessary for rod outer segment development through regulating ciliary protein trafficking. The *rpgrip1* mutant zebrafish may provide a platform for developing therapeutic treatments for RP patients.

## Introduction

Inherited photoreceptor dystrophies (IPD) are a group of genetically and phenotypically heterogeneous retinal diseases, characterized by the progressive death of photoreceptor cells^1^. Leber congenital amaurosis (LCA) is the most severe form of childhood IPD, causing early severe visual defects after birth^2^. LCA is also genetically heterogeneous with at least 17 genes implicated (2, the Retinal Information Network (RetNet) database). The *RPGRIP1* gene, encoding retinitis pigmentosa GTPase regulator interacting protein 1, was first identified as an RPGR interacting partner by three independent research groups^3–5^. Defects in the RPGR gene result in severe X-linked retinitis pigmentosa (XLRP), accounting for over 70% of XLRP cases^6^. *RPGRIP1* is a recessive LCA-causing gene that is mutated in 5% of all LCA cases^7,8^. Mutations in *RPGRIP1* also caused juvenile retinitis pigmentosa and cone-rod dystrophy in humans^9,10^. Similarly naturally occurring recessive mutations in *RPGRIP1* cause cone-rod dystrophy in dogs^11^.

RPGRIP1 localizes predominantly to the connecting cilium of mouse photoreceptor cells and to outer segments of human photoreceptors^12,13^. RPGRIP1 also localizes at the centrosome of non-ciliated cells and at the basal body of ciliated cells^14,15^. RPGRIP1 is a multi-domain protein, containing an N-terminal coiled-coil domain, two protein kinase C conserved domains (C2) and a highly-conserved C-terminal RPGR-interacting domain (RID)^16,17^. RPGRIP1 is reported to interact with different proteins. The N-terminal coiled-coil domain of RPGRIP1 interacts with SPATA7, which is mutated in LCA3 and juvenile RP^18,19^, the central C2 domain interacts with NPHP4 and notably disease-associated mutations in RPGRIP1 or NPHP4 disrupt this interaction^20^; the RID domain interacts with RPGR^3–5^ and Nek4 serine/threonine kinase^15^. RPGRIP1 is an anchor for RPGR localization to the connecting cilium^21^, but its targeting to the connecting cilium depends on SPATA7^18^. RPGRIP1 is also required for ciliary targeting of NPHP4 and SDCCAG8, which are associated with renal-retinal ciliopathy^22^. These data suggest RPGRIP1 plays a critical role in ciliary protein trafficking and ciliopathies presenting with the photoreceptor cell death.

An *RPGRIP1* knock-out (KO) mouse model exhibited early retinal degeneration with almost complete loss of photoreceptor cells by three months of age. The photoreceptors of these KO mice initially developed with a normal structure of the connecting cilium, but the outer segments were disorganized with oversized outer segment disks^21^. An ***N***-Ethyl-***N***-Nitrosourea (ENU)-induced RPGRIP1 null mouse model showed a more severe photoreceptor degeneration compared to the *RPGRIP1* KO mice^23^. The loss of the outer nuclear layer was initiated by postnatal day (P) 12 and nearly complete by P28. The rod outer segments were rarely formed, while the cone outer segments were initially formed but degenerated rapidly^23^. A naturally occurring dog model has a 44-nucleotide insertion in exon 2 of the *Rpgrip1* gene, resulting in an altered open reading frame and introduction of a premature stop codon^11^. RPGRIP1 deficient dogs exhibited loss of cone function by 6 weeks of age consistent with shortened inner and outer segments. Rod photoreceptor function appeared normal by 10 weeks of age, but by 23 weeks of age rod cells grossly degenerated with a significantly thinned outer nuclear layer. Only a few photoreceptors remained by 45 weeks of age^24^.

Here we report a new zebrafish model of RPGRIP1 generated by ethyl nitrosourea (ENU) mutagenesis. Homozygous *rpgrip1* mutant fish exhibited severely affected rod cells at an early stage, followed by progressive degeneration of cone cells. Rod outer segments were absent and rhodopsin mislocalization was apparent by 5 days post fertilization (dpf) of age, the earliest time point examined, as were trafficking defects of some ciliary proteins. Our data support the hypothesis that Rpgrip1 is required for rod outer segment formation possibly through regulating ciliary transport processes.

## Results

### Identification of zebrafish rpgrip1 mutants

**Figure 1:**
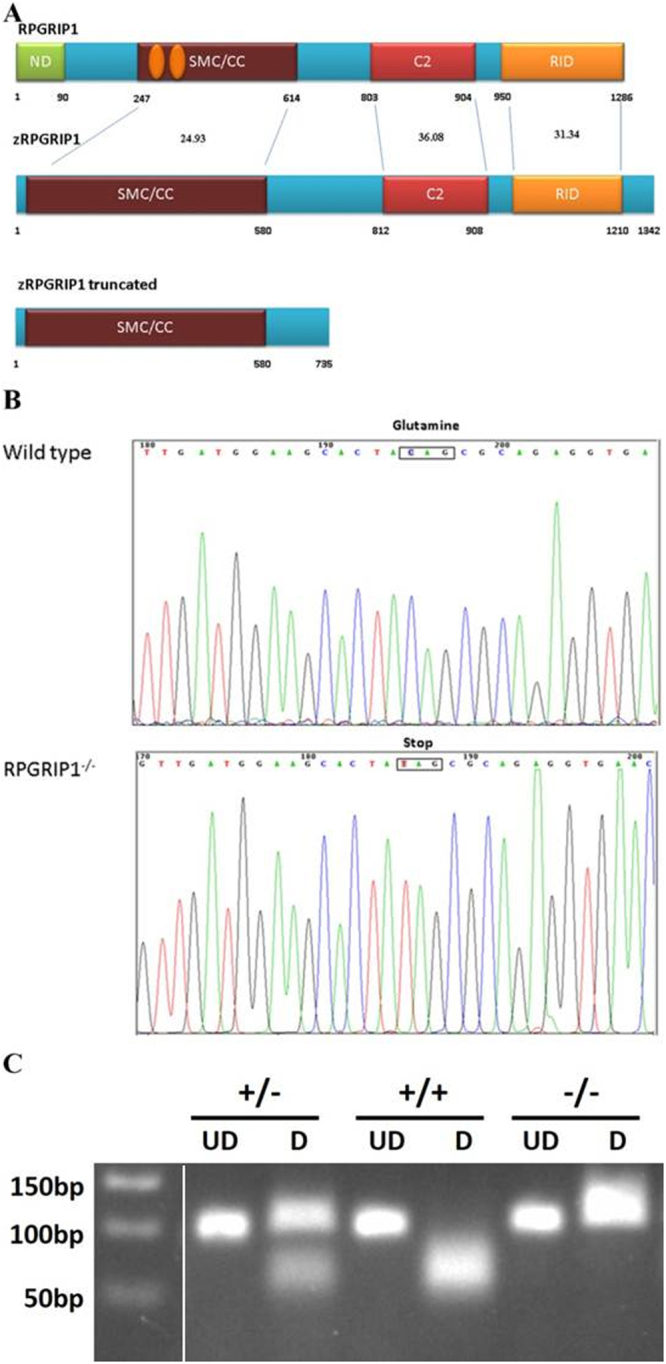
Genetic identification of *rpgrip1* mutant zebrafish. **(A)** Schematic structure of human RPGRIP1, zebrafish Rrpgrip1 (zRPGRIP1) and truncated zebrafish *rpgrip1* mutant. (**B)** Sequence validation of the C to T conversion in homozygous zebrafish, which resulted in a nonsense mutation and changing glutamine codon into stop codon (CAG to TAG, indicated with a box). (**C**) Gel picture of BbvI digested PCR products amplified using wild type and heterozygous fish genomic DNAs as templates. The PCR fragment was 99 bp and BbvI cut the fragment amplified from wildtype zebrafish to 59 bp and 40 bp, but could not cut the fragment amplified from mutant zebrafish. Wildtype zebrafish (*rpgrip1* ^+/+^) showed only one band because both bands (59 bp and 40 bp) are too close and could not be well separated. The heterozygous zebrafish, *rpgrip1* ^+/−^, showed two bands: the upper band is for the mutant PCR product, the lower band is BbvI-cut PCR product (59 bp and 40 bp). The homozygous mutant zebrafish, *rpgrip1* ^*−/−*^, showed one band, the 99 bp fragment, which BbvI could not cut. UD, undigested PCR product; D, BbvI-digested fragments.

Zebrafish *rpgrip1* encodes an open reading frame of 1342 amino acids and contains at least 28 exons, spanning approximately 28 kb on chromosome 24. Zebrafish Rpgrip1 protein has similar functional domains to human RPGRIP1with conserved protein identities of 24.93%, 36.08% and 31.34% for SMC/CC, C2 and RID domains, respectively (Fig. 1A). Zebrafish *rpgrip1* expression significantly increased during embryogenesis (Supplementary Material, Fig. S 1A and B), and in adult tissues *rpgrip1* was detected predominantly in the eye (Supplementary Material, Fig. S 1C and D). A large library of ENU-mutagenized fish were screened by TILLING^25^ for mutations in zebrafish ciliopathy genes, a C → T transversion at nucleotide 2206 in exon 18 of the *rpgrip1* gene was identified, resulting in a premature stop codon (Q736X) and loss of a *BbvI* restriction site, which was subsequently used for genotyping *rpgrip1* mutants (Fig. 1B,C). We predicted that the nonsense mutation would lead to either a truncated 735 amino acids polypeptide and/or nonsense-mediated RNA decay (NMD). Quantitative real-time PCR assay found *rpgrip1* mRNA was decreased by about 85% in 5 dpf *rpgrip1* ^−/−^ embryos when compared to wildtype siblings (Supplementary Material, Fig. S2), confirming that NMD was most likely occurring. Both heterozygous and homozygous *rpgrip1* mutant fish exhibited normal external morphology and were fertile. The mutant larvae also developed a normal swim bladder, an indirect sign of fish health. We phenotypically characterized the mutant fish, all the examined homozygous *rpgrip1* fish showed same phenotypes, suggesting a complete penetrance. There is no variable expressivity. Because zebrafish *rpgrip1* is a large gene, we could not clone the full-length cDNA, so we did not performed any rescue experiment.

### Rod outer segment development was defective in rpgrip1 mutants

**Figure 2:**
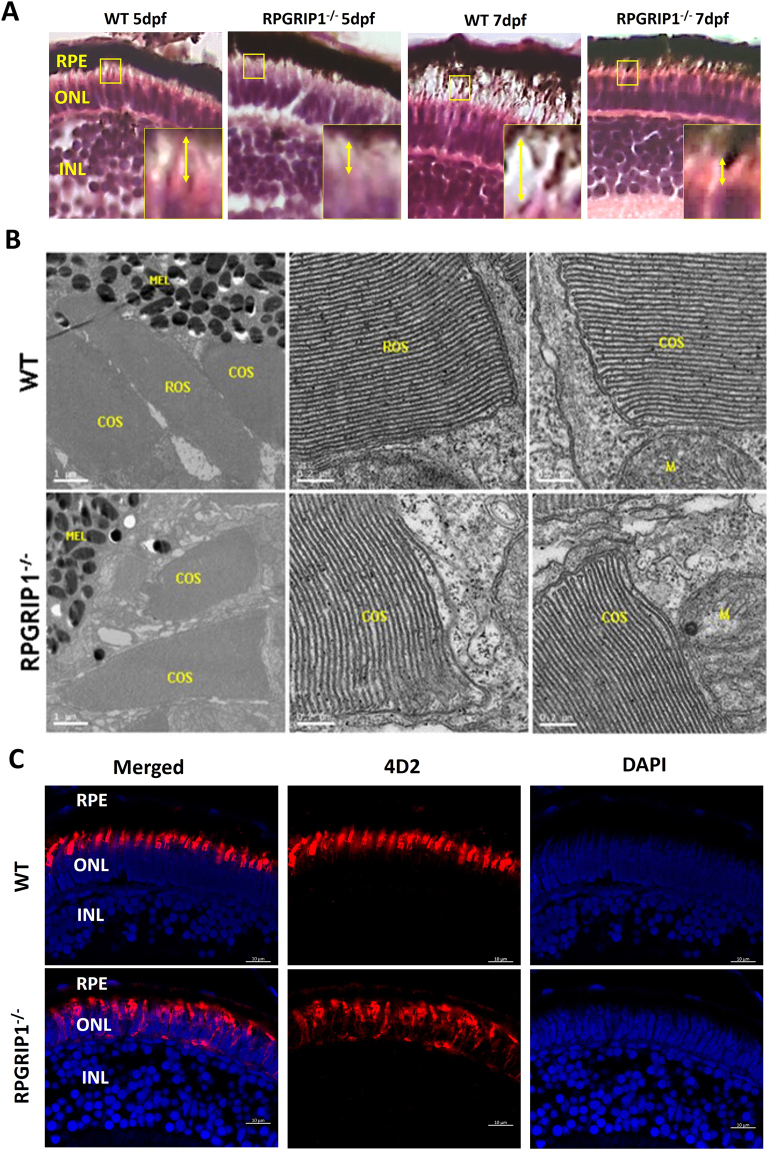
Rod outer segments were not developed in *rpgrip1* ^−/−^ zebrafish. (**A**) Photoreceptor outer segments and inner segments were shorter in the retinas of *rpgrip1* ^*−/−*^ zebrafish at 5 and 7dpf shown by haematoxylin & eosin staining. Double-headed yellow arrow shows the outer/inner segments; the measurement of outer segment length was shown in Supplementary Materials Fig. S3. Inserts show a 3.5 × magnification of the selected regions. (**B**) Ultrastructural analysis using transmission electron microscopy revealed both rod and cone outer segments were formed in the retinas of 5dpf wildtype siblings but only cone outer segments were presented in the mutants; rod outer segments were not observed in the retinas of 5 examined *RPGRIP1* ^*−/−*^ mutants at 5dpf. (**C**) Immunostaining with anti-rhodopsin antibody (4D2) further confirmed rod outer segments were not formed in the retinas of *rpgrip1* ^−/−^ mutants at 5dpf; rhodopsin was mislocalized in whole rod cell body. Nuclei were shown in blue using DAPI. COS, cone outer segments; GCL, ganglion cell layer; INL, inner nuclear layer; M, mitochondria; MEL, melanosome; ONL, outer nuclear layer; RPE, retinal pigment epithelium; ROS, rod outer segments.

Previous reports in mice suggested RPGRIP1 participates in rod outer segment formation and disk morphogenesis^21,23^. We first examined photoreceptors in wildtype, heterozygous and homozygous *rpgrip1* mutant larvae at 5 and 7 dpf by hematoxylin and eosin staining. Photoreceptor outer/inner segments of wildtype, heterozygous and homozygous siblings at 5 and 7 dpf were visible by light microscopy of retinal sections(the *rpgrip1* ^+/−^ larvae were indistinguishable from wildtype siblings - data not shown); however, outer/inner segments were significantly shorter in *rpgrip1* ^−/−^ retina compared to wildtype siblings (Fig. 2A and Supplementary Material, Fig. S 3). It is well known that rod outer segments are longer, thinner and more cylindrical in shape compared to cone outer segments which are shorter and conical shaped^26–28^. Rod outer segments are surrounded by plasma membrane; whereas cone outer segments have parallel membranes called lamellae, which are partially closed on one side by plasmalemma^26–28^. To determine whether the significantly decreased length of outer/inner segments in *rpgrip1* mutant larvae was caused by the defect of rod outer segment development, we examined outer segment ultrastructure in wildtype and *rpgrip1* mutant siblings using transmission electron microscopy. At 5dpf rod and cone outer segments were well formed in wildtype siblings; whereas in *rpgrip1* ^−/−^ mutants rod outer segments were not observed, however, cone outer segments were normal (Fig. 2B). Anti-rhodopsin antibody (4D2) labelling of rod outer segments by immunofluorescent analysis demonstrated stained outer segments visible in wildtype but absent in *rpgrip1* ^*−/−*^ siblings. Rhodopsin was mislocalized in rod cells through to the synapses (Fig. 2C).

### Early retinal degeneration in rpgrip1 mutants

**Figure 3:**
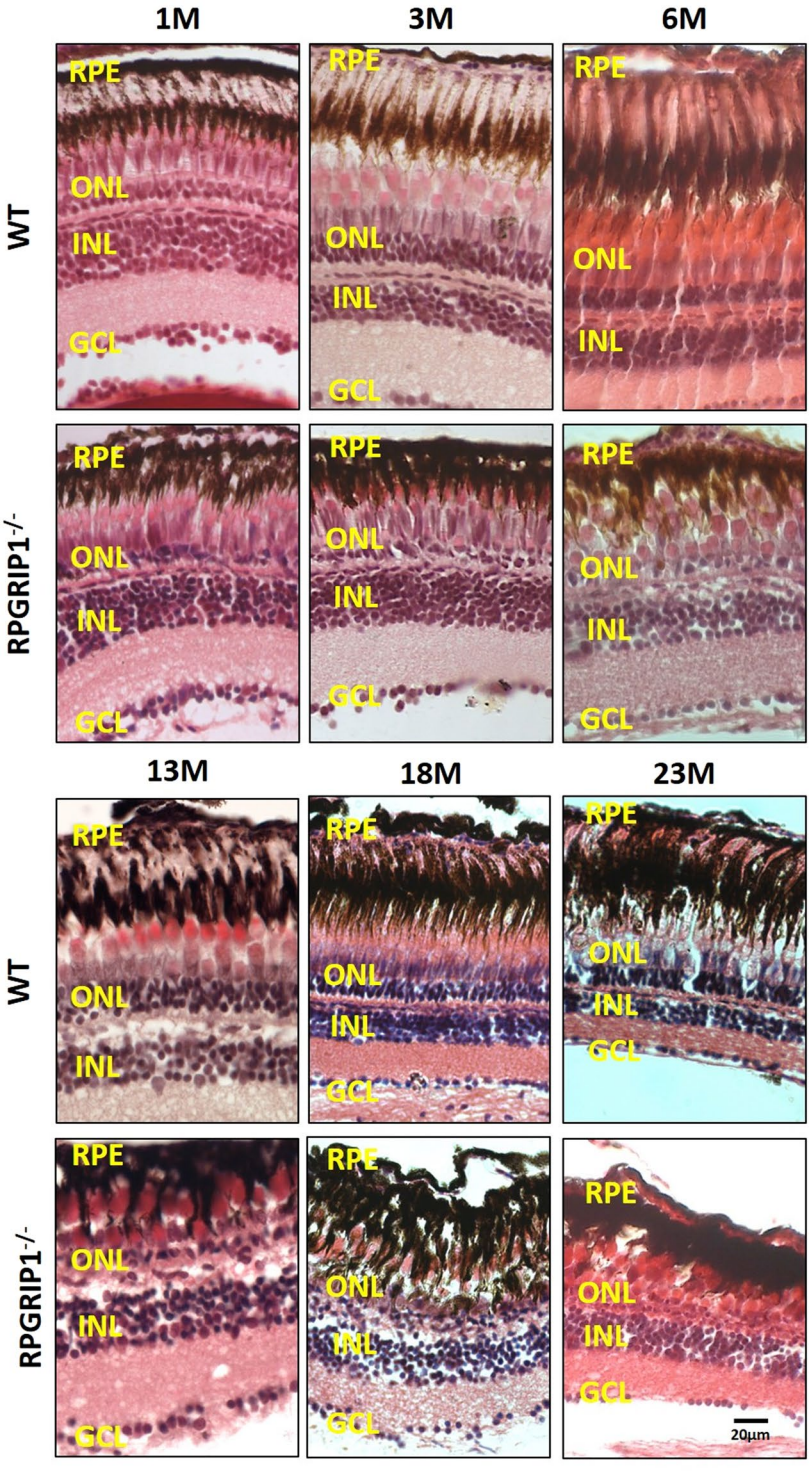
Haematoxylin & eosin staining of retinal sections of wild type and *rpgrip1* ^−/−^ zebrafish at different ages, showing progressive retinal degeneration. WT, wildtype; GCL, ganglion cell layer; INL, inner nuclear layer; ONL, outer nuclear layer; RPE, retinal pigment epithelium. The scale bars are 10 µm.

**Figure 4:**
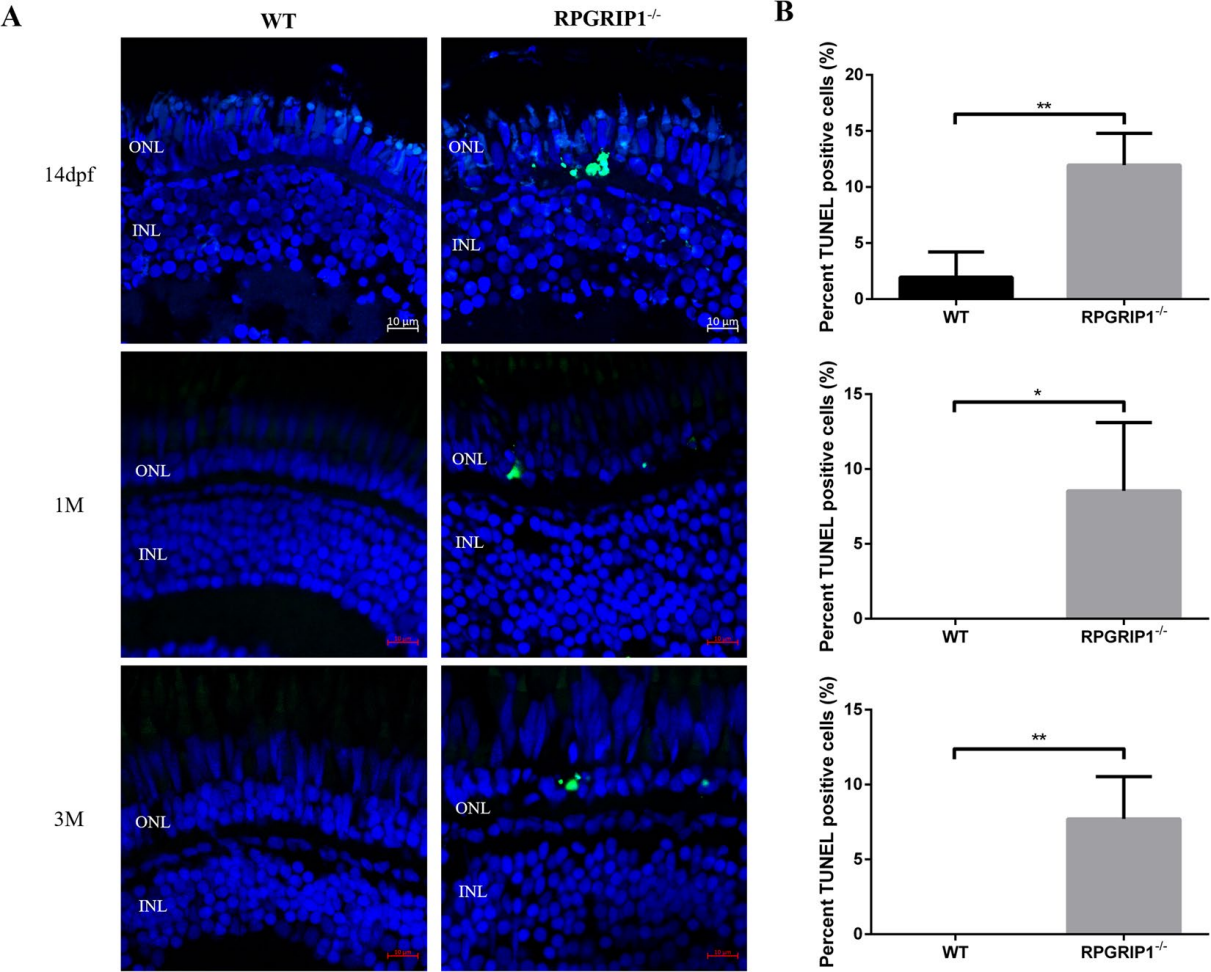
TUNEL assay showed significantly increased cell death in *rpgrip1* ^−/−^ mutant retinas. Retina cryosections of wildtype (WT) and *rpgrip1* ^−/−^ zebrafish at 14 dpf, 1 mf and 3 mpf were subjected to TUNEL assay. (**A**) TUNEL-positive cells were present in the outer nuclear layer (ONL) of *rpgrip1* ^−/−^ mutant retinas. TUNEL-positive cells were barely observed in ONL of wildtype retina at three age points. (**B**) Graph indicating significant cell death (12%, 9% and 8% respectively) in the mutant retina compared to the wildtype retina at each age point. The data were collected by counting positive cells from 5 retinal sections of wildtype and rpgrip1 mutant respectively and shown as mean ± SD. SD: standard deviation. **p < 0.01.

Previous reports show that RPGRIP1 mutations can cause LCA, juvenile RP and cone-rod dystrophy in humans^7–10^ and early-onset retinal degeneration in mice^21,23^. We examined retinal degeneration in heterozygous and homozygous fish at different ages using histological methods. Heterozygous fish did not present with photoreceptor abnormalities, even as late as 24 months of age (data not shown). The outer nuclear layers of *rpgrip1* ^−/−^ retinas at 5 and 7 dpf were the same as that of wildtype siblings (Fig. 2A); however, some rod cells displayed an abnormal morphology with condensed and rounded-up nuclei at 2 weeks of age. By age of 3 weeks, degeneration of rod cells was evident in the mutant fish (data not shown). Only some rod nuclei remained at 1 month post-fertilization (mpf) when compared to age-matched controls (Fig. 3). Zebrafish retina development continues into adult stages^29^, as observed in 3 mpf controls, where approximately four layers of rod nuclei were visible, but in the *rpgrip1* mutant, only a few rod nuclei were present. This is consistent with the loss of photoreceptor cells, and in agreement we detected significantly increased cell death in *rpgrip1* mutant retinas at 14 dpf, 1 mpf and 3 mpf using TUNEL assays (Fig. 4). At 3 mpf, cone cells presented normal morphology, though they were not tightly aligned together as in wildtype siblings (Fig. 3), possibly due to the degeneration of the rod cells resulting in structural changes to the cone cell layer. At 6 mpf, the cone cells appeared disorganized and loss of cone cells was much more evident in *rpgrip1* ^−/−^ mutants. At 13 mpf, the number of cone cells was further decreased (Fig. 3). Transmission electron micrographs of the retinal sections of *rpgrip1* ^*−/−*^ mutants at 13 mpf showed that only single cones with normal morphology remained (Supplementary Material, Fig. S4). At 18 mpf single cones were also affected: the morphology of these cells was abnormal and some were degenerated (Fig. 3); At 23 mpf very few single cone cells were evident (Fig. 3).

**Figure 5:**
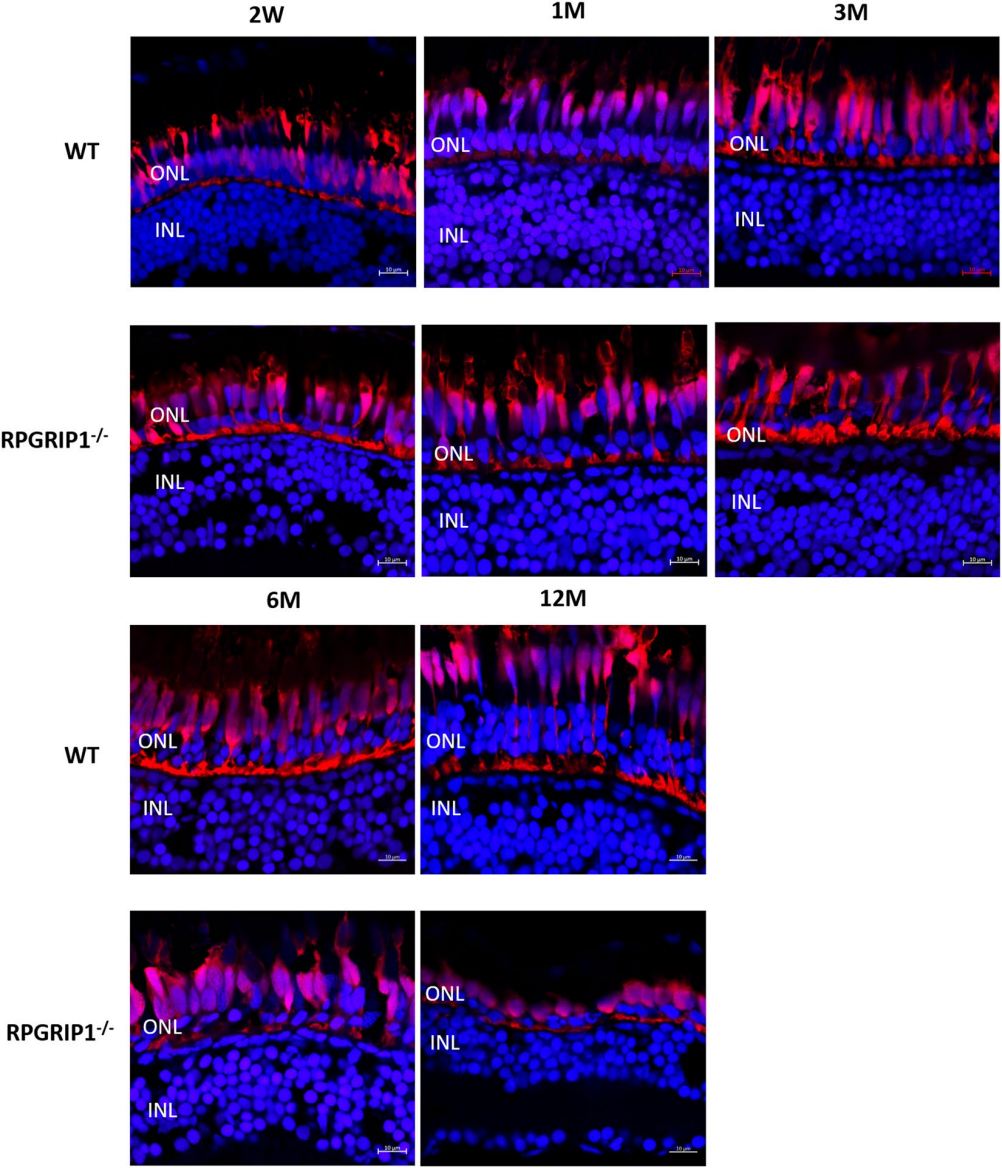
Immunostaining with Zpr1 antibody exhibited red/green double cones that were normal at early ages and degenerated at later ages. Nuclei were shown in blue using DAPI. Double cones of *rpgrip1* ^−/−^ mutants were morphologically normal at 2wpf and 1 mpf, shown here. While at 3mpf, double cones were disorganized, the number of double cones was markedly reduced at 6 mpf and only some double cone cell bodies remained at 12 mpf.

**Figure 6:**
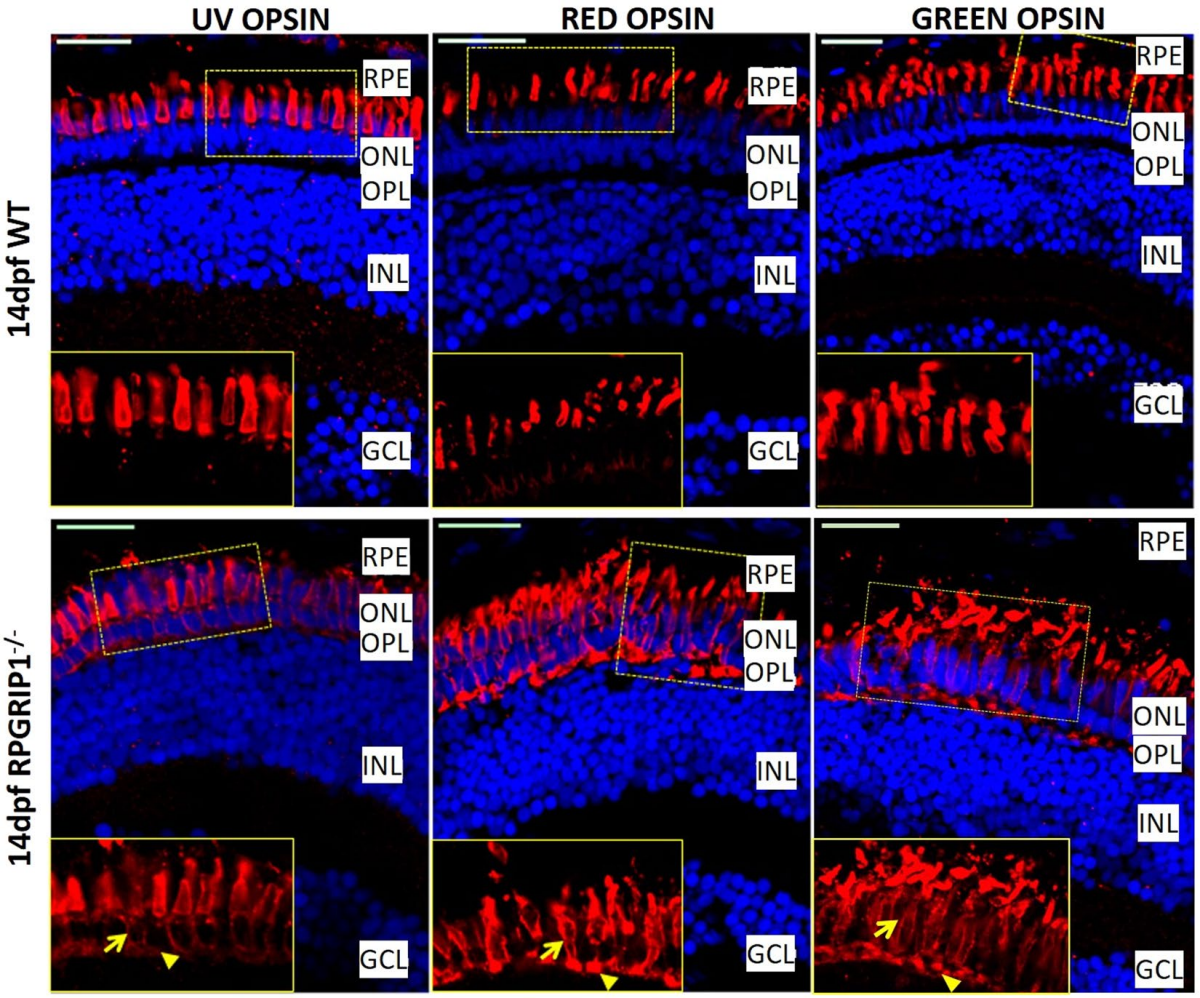
Partial mislocalization of cone opsins in *rpgrip1* ^−/−^ retinas. Green, red and UV opsins were localized in cone outer segments of 14 dpf wildtype zebrafish retinas. In *rpgrip1* mutant zebrafish retinas, green, red and UV opsins were localized in outer segments and partially mislocalized in cone cell bodies (arrow) and synapses (arrowhead). GCL, ganglion cell layer; INL, inner nuclear layer; ONL, outer nuclear layer; OPL, outer plexiform layer; RPE, retinal pigment epithelium.

Immunofluorescence labelling was used to examine both rod and cone cells. Rhodopsin was mislocalized to the inner segments, outer nuclear layer and synapses of rod cells at all time points examined (Fig. 2C and Supplementary Material, Fig. S5). The rod cells appeared to be disorganized at 14 dpf. The fluorescence signal (labelled by 4D2 antibody) was remarkably reduced by 1 mpf, suggesting significant degeneration of rod cells. At 3 mpf, only a weak fluorescence signals were detected, possibly because all the rod cells had degenerated. At 6 mpf, only a little background fluorescence signals were presented (Supplementary Material, Fig. S5). Immunostaining with ZPR1 antibody which labels red and green cone cells (double cones) showed those cells were morphologically normal at 3, 4, 5 dpf and 1 mpf (Fig. 5 and data not shown). At 6 mpf, the cone cells were markedly disorganized and the outer segments were obviously shorter. By 12 mpf, only a few fragmented double cone cell bodies were observed (Fig. 5). As cone opsins are partially mislocalized in the cell bodies of *rpgrip1*mutant mice^21,23^, we examined cone opsin localization in the retinas of *rpgrip1* ^−/−^ and wildtype siblings at 14 dpf using antibodies which target to red, green or UV opsins. Red, green and UV opsins were localized in cone outer segments in the wildtype retina; however, the three types of opsins were partially mislocalized in the outer nuclear layer of *rpgrip1* ^−/−^ mutants (Fig. 6).

### Visual function was impaired in RPGRIP1 mutants

**Figure 7:**
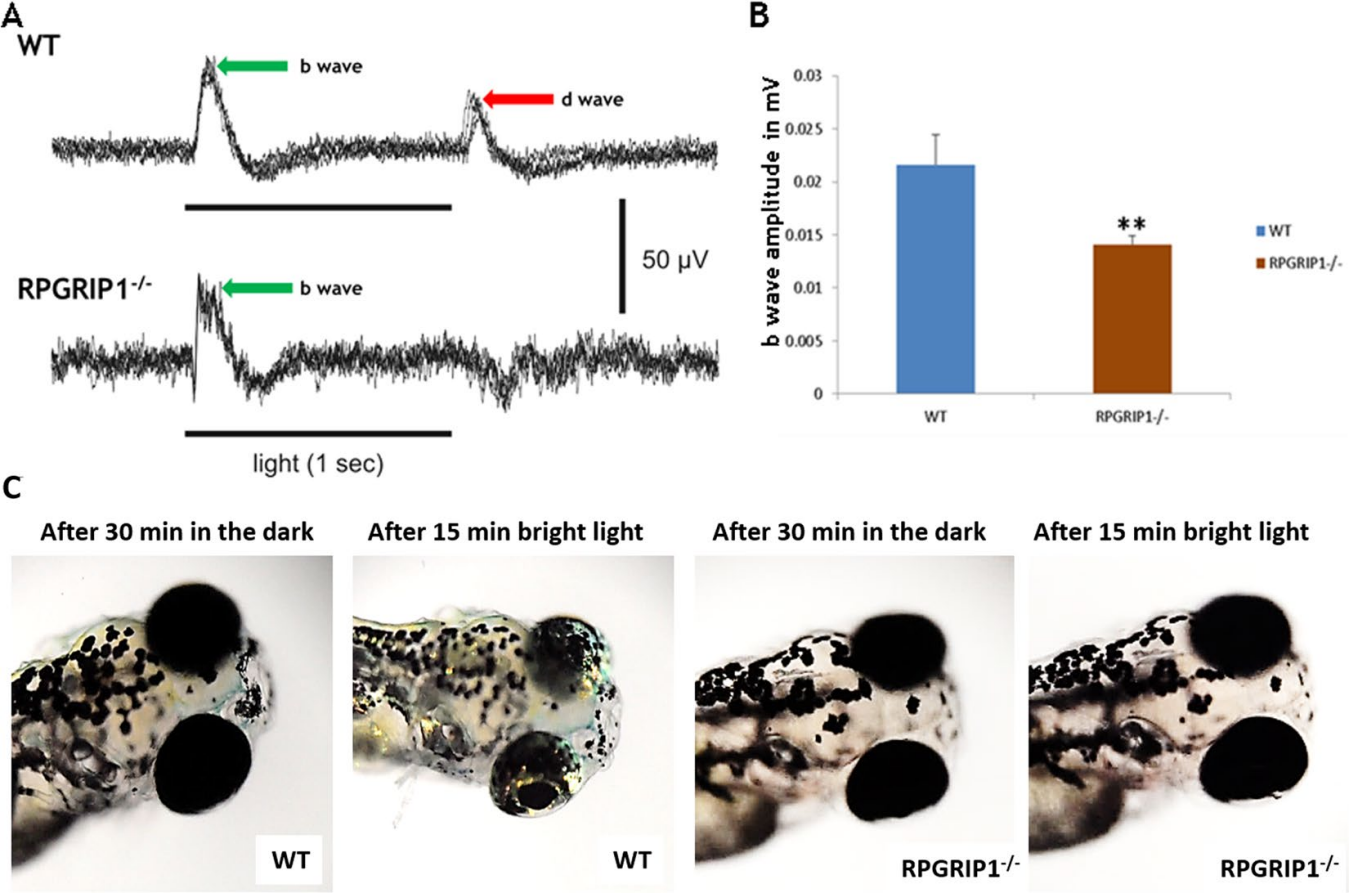
Impairment of visual function in *rpgrip1* ^−/−^ zebrafish. (**A**) Representative ERG traces from wildtype and *rpgrip1* ^−/−^ zebrafish at 7dpf. D-wave was absent in the mutant zebrafish. (**B**) b-wave amplitudes of *rpgrip1* ^*−/−*^ zebrafish (n = 10) were significantly reduced when compared to wildtype siblings (n = 11). The result is shown as mean ± SD. SD: standard deviation. **p < 0.01. (**C**) Visual background adaption for wildtype (n = 10) and *rpgrip1* ^−/−^ zebrafish (n = 10) at 7dpf responding to dark adaption and bright light stimulation. Representative images showing that melanin of wildtype zebrafish was aggregated into small granules after light adaption, while *rpgrip1* ^−/−^ zebrafish displayed larger diffused melanin granules.

We used electroretinography (ERG) recordings to evaluate *rpgrip1* ^*−/−*^ fish for abnormal visual function. Zebrafish cone cells mature earlier than rod cells, so the ERG response of larvae at 5 and 7 dpf is determined by cones^30,31^. 7dpf zebrafish were dark-adapted for at least 30 minutes before ERG recordings. Compared with wildtype siblings, the scotopic b-wave amplitudes were significantly reduced by approximately 30% (p < 0.01) and the OFF response (d-wave) was absent (Fig. 7A,B), suggesting dysfunction of cone cells, possibly due to the partial mislocalization of cone opsins (Fig. 6). We also evaluated whether *rpgrip1* ^*−/−*^ fish are functionally responsive to light by examining the visual background adaption (VBA) response in 7dpf fish. VBA, a neuroendocrine response, is controlled through the retino-thalamic tract^32^ and blind fish fail to display this response^33^. When fish are placed on a dark background, the pigment granules (melanosome) are dispersed within pigmented skin cells; in contrast when fish are placed on a light background, the melanosomes are aggregated to form small granules^32^. 7 dpf wildtype and *rpgrip1* ^−/−^ larvae were dark-adapted for 30 mins then exposed to bright light for 15 mins. The wildtype larvae exhibited small pigment granules as expected, however *rpgrip1* ^*−/−*^ larvae displayed larger diffuse pigment granules, suggesting visual impairment (Fig. 7C).

### Rpgrip1 mutants exhibited defects in ciliary protein trafficking

Mammalian RPGRIP1 directly or indirectly interacts with different proteins and possibly functions in ciliary protein trafficking^17^. RPGRIP1 physically interacts with RPGR through its C-terminal RPGR-interacting domain^3–5^. Localization of RPGR to the connecting cilium of mouse retina is dependent on RPGRIP1, and RPGR was absent in the connecting cilium of RPGRIP1 knock-out mouse^21^. We examined whether Rpgr localization in *rpgrip1* ^−/−^ zebrafish retina was affected. Immunostaining with an antibody, previously used to localize RPGR to connecting cilia and outer segments of adult zebrafish photoreceptors^34^, revealed that Rpgr was localized to photoreceptor inner segments of 14 dpf *rpgrip1* ^−/−^ zebrafish retina, while Rpgr was localized to outer segments and connecting cilia of wildtype sibling photoreceptors. Rpgr signal was also significantly decreased in *rpgrip1* mutant retinas (Supplementary Material, Fig. S 6A).

**Figure 8:**
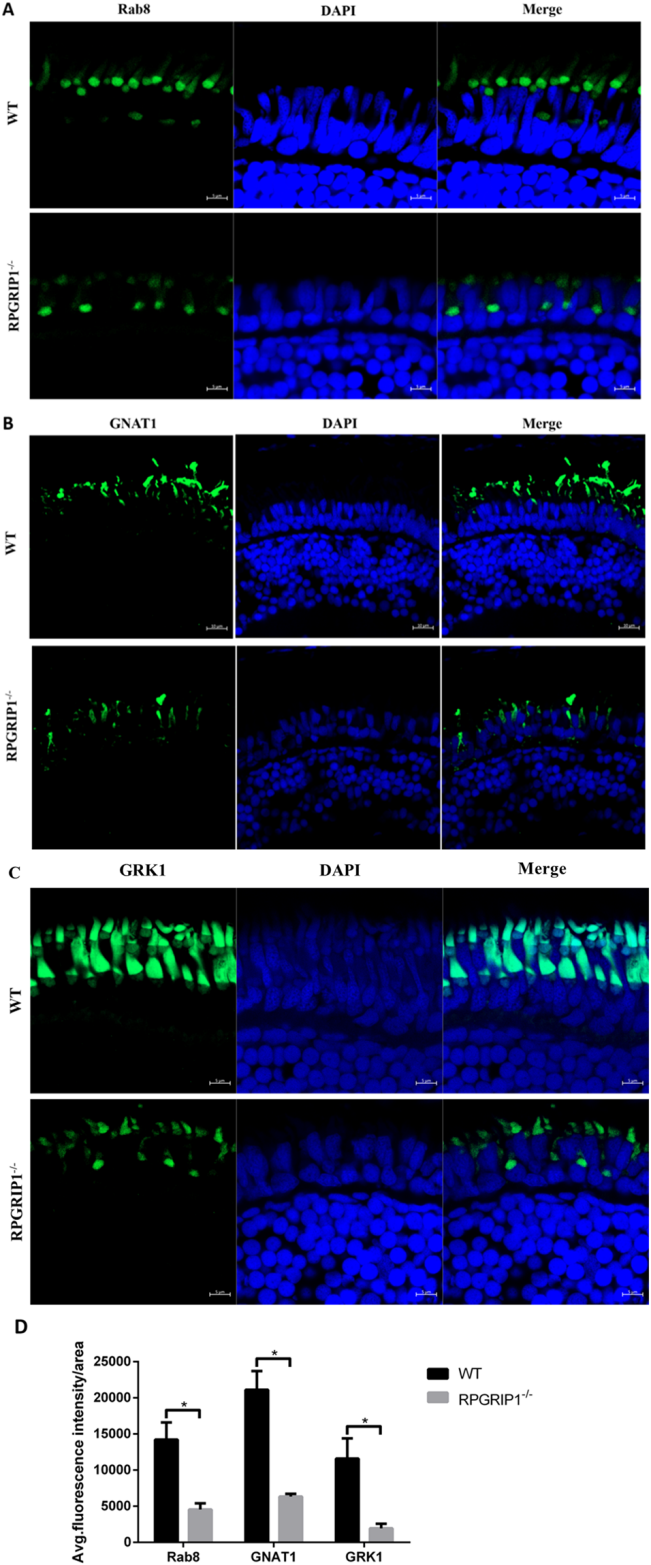
Mislocalization of ciliary proteins in *rpgrip1* mutant fish. (**A**) Rab8 was mainly localized to the connecting cilium of rod cells and some weak fluorescence signals were present in the outer segments of photoreceptor cells in wildtype (WT) zebrafish at 2 wpf; it was mislocalized in inner segments of photoreceptor cells and the localization to the outer segments of photoreceptor cells was lost in *rpgrip1* mutant zebrafish at 2 wpf. (**B**,**C**) Transducin and GRK1 exhibited abnormal distribution in cone outer segments and loss of localization to rod outer segment, because the rod outer segments were absent in *rpgrip1* mutant zebrafish. INL, inner nuclear layer; ONL, outer nuclear layer. (**D**) Quantification of fluorescence intensity of Rab8, GNAT1 and GRK1 signals. The fluorescence intensity was measured within three outer segment areas (25 × 25 µm²/area) in each section (four sections from four individual retinas were used) by Zen (Zeiss) at the condition of same laser intensity and master gain, and the data was analysed by t test with Graph Prism. The result is shown as mean ± SD. SD: standard deviation. *p* = 0.0027 (Rab8); *p* = 0.0084 (GNAT1); *p* = 0.0164 (GRK1).

Previous work showed RPGR directly interacted with a small GTPase Rab8 and functioned as a guanine nucleotide exchange factor (GEF) for Rab8^35^. Knock-down of RPGR reduced Rab8 targeting to primary cilia *in vitro* ^35^. Since RPGR localization was disrupted in *RPGRIP1* knockout mouse^21^, we asked whether Rab8 localization in *rpgrip1* ^−/−^ fish photoreceptors was affected. Immunofluorescence assays were performed using an anti-Rab8 polyclonal antibody raised against a human Rab8 peptide from the C-terminal end that is highly conserved between human and zebrafish Rab8 (86% identity, data not shown). Rab8 was mainly localised to the ciliary base of photoreceptor cells, though weak signal was also detected in outer segments of photoreceptor cells (Fig. 8A), consistent with early reports of Rab8 localization in zebrafish, *Xenopus* and mouse photoreceptors^36–39^. However in *rpgrip1* ^−/−^ siblings, the Rab8 signal at the ciliary base was significantly decreased, and signals were also detected in the inner segments of cones, the localization of Rab8 to the outer segments of photoreceptor cells was also lost, indicating RPGRIP1 is partially required for the Rab8 localization in photoreceptors (Fig. 8A,D).

Transducin has been reported to be mislocalized in *Rpgrip1* ^*nmf247*^ mutant mice^23^. We wondered whether transducin was mislocalized in *rpgrip1* ^−/−^ zebrafish. We examined transducin localization in zebrafish retina by immunostaining with anti-Gnat1 (the alpha subunit of rod transducin) antibody, which was used for immunofluorescence assay in *rp2* knock-out zebrafish^40^. At 14 dpf, Gnat1 was localized at the outer segments of wildtype photoreceptors. However, the fluorescence signal of Gnat1 was mainly observed in the inner segments of rod cells of *rpgrip1* ^−/−^ mutants, possibly because rod outer segments were absent, and the fluorescence signals were also significantly reduced (Fig. 8B,D). The localization of G-protein-coupled receptor kinases (GRK1 and GRK7) in wildtype and *rpgrip1* mutant retinas was examined using anti-human GRK1 and anti-zebrafish GRK7a antibodies^41,42^. GRK1 was detected in outer segments of both rod and cone cells, probably because the anti-human GRK1 antibody could recognize both zebrafish GRK1a and GRK1b, which were localized to rod and cone outer segments respectively^42^. However, GRK1 signals were localized at inner segments of rod cells and most GRK1 fluorescence signals were significantly reduced from cone outer segments (Fig. 8C,D). Additionally, the localization of GRK7a (the cone opsin kinase) was not affected in *rpgrip1* ^−/−^ mutants (Supplementary Material, Fig. S6B,C). Since the fluorescence signals of RPGR, rab8, GNAT1, GRK1 were decreased in rpgrip1 mutant retinas at 14 dpf, we examined the level of these proteins and found expression of all proteins to be significantly decreased (Supplementary Material, Fig. S7).

## Discussion

In this study, we characterized a newly identified zebrafish mutant, which carried a nonsense mutation in the *rpgrip1* gene. *Rpgrip1* ^−/−^ zebrafish did not form rod OS and displayed rhodopsin mislocalization. However, cone cells developed normally, although cone opsins were partially mislocalized, suggesting RPGRIP1 may have a major role in rod cells. Mice with complete depletion of *rpgrip1* also do not form rod OS, but cone OS was initially formed and abnormal^23^. In contrast to zebrafish and mouse mutants, *rpgrip1* mutant dogs appeared to form both rod and cone OS^24^. The vertebrate photoreceptor OS is a specialized compartment, comprising stacks of disk membranes. It contains the visual pigment molecules essential for phototransduction. Rhodopsin is the most abundant molecule contained within OS membranes and appears to be required for OS elaboration. Mice carrying homozygous mutations in the *Rho* gene do not form OS^43^. Depletion of the transcription factor, CRX, which regulates expression of many retinal genes including the *Rho* gene, also results in a failure of OS formation in the mouse model^44^.

Rhodopsin is synthesized in the endoplasmic reticulum and undergoes glycosylation in the Golgi complex. After sorting and exiting from the Golgi complex, rhodopsin reaches the trans-Golgi network (TGN) membranes and incorporates into rhodopsin transport carriers (RTCs), where it binds the activated small GTPase Arf4 through its C-terminal VxPx ciliary targeting signal to initiate the assembly of the rhodopsin ciliary targeting complex. Rab8 is activated by Rabin8 during budding of RTCs and in turn this regulates the tethering of RTCs to the periciliary plasma membrane and fusion with the ciliary membranes. Once rhodopsin is in the ciliary membranes, it is further transported to rod OS through motor proteins and intraflagellar transport machinery^45,46^. Defects in any step of the trafficking procedures will result in rhodopsin mislocalization, abnormal rod OS formation and retinal degeneration. Rab8 is a major participant of rhodopsin-bearing vesicle trafficking and play a critical role for the delivery of rhodopsin-containing post-Golgi vesicles to the base of the connecting cilium. Early data showed Rab8 was enriched in post-Golgi vesicles, co-localized with rhodopsin-containing post-Golgi vesicles at the base of OS, and also localized at the ciliary stalk of OS. Rab8 was also associated and partially colocalized with microfilaments^37^. Transgenic *Xenopus* carrying the canine Rab8 dominant negative (T22N) form display rhodopsin mislocalization and accumulation of rhodopsin-bearing vesicles near the periciliary ridge complex, indicating Rab8 is required for docking the post-Golgi rhodopsin-containing membrane to the plasma membrane^38^. However, recent data demonstrated that mice with deletion of Rab8a only or with Rab8a deletion and overexpression of Rab8b dominant-negative form had normal outer segment structure and correct distribution of rhodopsin and other phototransduction proteins, suggesting Rab8 is not required for mouse rhodopsin targeting to outer segments^47^. However, the current findings in our study indicate that RPGRIP1 is required for Rab8 ciliary localization and is involved in rhodopsin ciliary vesicle transport in zebrafish.

The mechanisms underlying the role of RPGRIP1 for Rab8 ciliary localization remains elusive. It is possible that this is through RPGR or other RPGRIP1 interacting partners. CEP290, a component of the RGRIP1 protein complex and a ciliopathy-causing gene, is also required for Rab8 primary cilium localization^48^. CC2D2A, a CEP290 interactor, also has a role in Rab8 mediated ciliary vesicle transport, and deficiency of Cc2d2a in zebrafish leads to mislocalization of Rab8 in cone cells and abnormal OS development^36^. Depletion of another ciliopathy-causing gene, *Ahi1*, also a CEP290 interactor, in mice resulted in decreased Rab8 at the base of cilium^39^. The Bardet-Biedl syndrome (BBS) protein complex (BBSome) mediates ciliogenesis by recruiting Rab8 to the primary cilium through BBS1 physically interacting with Rabin8^49^. The ciliopathies are a group of highly genetically heterogeneous disorders and the ciliopathy-associated proteins form a protein complex/network, e.g. BBSome and NPHP-JBTS-MKS, for their interlinked function^49,50^. It is highly possible that the ciliopathy protein complex/network may share some common molecular pathways, one of which is Rab8 mediated post-Golgi ciliary vesicle trafficking.

Rhodopsin mislocalization is a common phenotypic feature in humans and animal models with retinal ciliopathy. RPGRIP1 deficiency in mouse, dog and zebrafish causes rhodopsin mislocalization (Fig. 2C, Supplementary Material, Fig. S5, and ref.^21,23,24^). Similarly, depletion of two RPGRIP1 physically interacting partners, NPHP4 and SPATA7, in mice also results in mislocalization of rhodopsin^18,20^. It has been proposed that mislocalized rhodopsin contributes to photoreceptor cell death, that the level of mislocalized rhodopsin most likely correlates to the speed of retinal degeneration and that the reduction of mislocalized rhodopsin can ameliorate rod cell death^18,51,52^. Mislocalized rhodopsin triggered the apoptosis of rod cells in the retinas of Spata7 and Kif3a mutant mice^18,52^. It is probably that rod death in *rpgrip1* ^−/−^ zebrafish is via apoptosis, which was confirmed by TUNEL assay (Fig. 4). How mislocalized rhodopsin triggers rod cell apoptosis remains to be determined, although an *in vitro* study has suggested rhodopsin mislocalization stimulates G proteins that increase the activity of adenylate cyclase thereby enhancing generation of cAMP^53^. Previous studies have reported that the cAMP level was increased in some RP animal models and, as a result, it has been proposed to be a promoter of photoreceptor cell apoptosis^54^.

In RP and LCA patients and animal models, loss of rod cells leads to secondary death of cones (1). *Rpgrip1* ^−/−^ zebrafish recapitulate the clinical presentations of LCA and juvenile RP patients by displaying early rapid rod cell degeneration followed by secondary cone cell death (Fig. 3 and Supplementary Material, Fig. S4). Zebrafish have a cone-rich retina with four types of cones (red, green, blue and UV) that provide an excellent model to study the mechanisms of cone cell death. In *rpgrip1* mutant zebrafish long cones (red and green cones) were fully degenerated by 13 months of age; short cones (blue and UV cones), however, appeared to be morphologically normal at that age (Supplementary Material, Fig. S4). Short cones were abnormally organized at 18 months of age and most single cones were degenerated by the age of 23 months. The mechanisms underlying selective cone cell death at different time points require further investigation.

In summary, the present study describes the cellular and molecular pathology of a novel *rpgrip1* mutant zebrafish model. This model will provide an opportunity for future investigation into the cellular function of RPGRIP1 and elucidation of the underlying disease mechanisms as well as enabling development of drug candidates for the treatment of conditions caused by *RPGRIP1* mutations.

## Materials and Methods

### Animals

An ENU (N-Nitroso-N-ethylurea) mutagenized library of F1 male zebrafish was prepared and ENU-induced heterozygous mutations were identified using CEL1-based TILLING (Targeting Induced Local Lesions IN Genomes)^25^. Zebrafish containing mutations were maintained for further characterization. All experiments using zebrafish were carried out in accordance with the UK home office animal care guidelines and approved by the Animal Ethics and Welfare Committee, Department of Life Sciences, Glasgow Caledonian University; a minimal number of animals were used for this study. Zebrafish larvae and adult were maintained at 28 °C on a 14/10 h light/dark cycle.

### Bioinformatic analysis

Peptide sequences of human and zebrafish RPGRIP1 were obtained from Ensembl.org database and were aligned using Clustal Omega alignment tool (http://www.ebi.ac.uk/Tools/msa/clustalo/). Conserved sequences were boxshaded using BoxShade 3.21 (http://www.ch.embnet.org/software/BOX_form.html) and primers were designed using NCBI primer design tool (http://www.ncbi.nlm.nih.gov/tools/primer-blast/).

### Gene expression analysis

Total RNA was extracted from different tissues of adult zebrafish and embryos at various stages of development using Trizol (Invitrogen) and cDNAs were synthesized using Trancriptor high fidelity cDNA synthesis kit (Roche). End-point PCR analysis was carried out using NEB standard Taq polymerase system and quantitative PCR was performed using platinum SYBR green qPCR supermix (Life technologies). The sequences of all the primers will be provided as requested.

### Genotyping

The zebrafish were genotyped using DNA extracted from the fin clip. Individual fish were anaesthetised by immersing in 0.016% Tricaine for 30–45 seconds, then a small portion of the tail fin was clipped and placed in a sterile 0.2 ml PCR tube. Once the fin was clipped the fish was immediately placed in system water to recover. To extract DNA from the fin clip, 25 µl of methanol was added and incubated at room temperature for 5 minutes. 15 µl of TE-Tween20 (10 mM Tris, 1 mM EDTA (pH 8.0), 0.33% Tween 20) and 0.5 µl of 10 mg/ml proteinase K were added to the sample and incubated at 56 °C for at least 2 hours in a thermal cycler machine. The tube was then incubated at 98 °C for 10 minutes to inactivate the proteinase K. 135 µl of dH_2_O was added to the sample and mixed well. 2 µl of the extracted DNA was used as template to amplify the DNA fragment using primers GT211: AAAAATGTACAAAAACTAAGGCCTACC and GT212: AAGAGCGAGGATCTCGTTGATGGAAGCACTG. The PCR products were subjected to sequencing or restriction digestion using *BbvI* restriction enzyme. 3 µl of the PCR sample was added to 1 µl of 10X NEB buffer 2, 0.5 µl *BbvI* (10U/µl) and 5.5 µl dH_2_O, and incubated at 37 °C for 3 hours and the samples were subjected to electrophoresis on 3% agarose gel at 70 V for 90 minutes.

### Histology and immunostaining

Enucleated eyes were fixed in 4% paraformaldehyde overnight. For haematoxylin & eosin staining and immunohistochemistry analysis, fixed eyes were dehydrated with series ethanol treatment and embedded in paraffin. For immunohistostaining, fixed eyes were washed in PBS twice (5 minutes each) and cryoprotected in 5%, 15% and 20% sucrose each for 2 hours (h) at room temperature (RT) and moulded in OCT medium (VWR) and 10 µm sections were cut in cryostat. 7 µm paraffin sections were deparaffinised and blocked in 2% BSA/PBS for 30 minutes at RT and then incubated in primary antibodies at RT for 2 h (Anti-rhodopsin (1:500; 4D2 from Abcam). After washing in PBS twice, sections were incubated at RT for 2 h in anti-FITC mouse (1:500; Sigma) diluted in 2% BSA/PBS. For immunostaining with anti-RPGR (1:500; Sigma, HPA001593), anti-Zpr1 (1:500; ZIRC), anti-Rab8 (1:500; Sigma), anti-GRK1 (1:50; Abcam), anti-GRK7a (1:50; gift from Prof. Stephan Neuhauss, University of Zurich) and anti-GNAT1 (1:50; Abcam) antibodies, 10 µm cryosections were thawed at RT for 30 minutes, blocked in 5% sheep serum, 0.3% Triton X-100 in PBS for 1 h and incubated in primary antibody diluted in diluting solution (1% BSA/PBS, 0.3% Triton X-100) for 2 h at RT. Then sections were washed with PBS thrice (5 minutes each) and then incubated in secondary antibodies for 2 h at RT (anti-FITC mouse, 1:500; Sigma), AlexaFluor 594-conjugated anti-rabbit (1:500; Invitrogen)). After incubating sections in the secondary antibody, the sections were washed with PBS twice (5 minutes each) and mounted with vectashield mounting medium containing DAPI, which stains the nuclei. All the sections were imaged using LSM 510 confocal microscopy (Carl Zeiss).

### TUNEL assay

Cell death in the retinas of *rpgrip1* ^−/−^ mutant zebrafish at 14 dpf, 1 mpf and 3 mpf was detected by the DeadEnd™ Fluorometric TUNEL System (Promega) following the manufacturer’s instruction. The cryosections of zebrafish eyes were washed with 1 × PBS for 5 min before being fixed again by 4% PFA/PBS. After permeabilisation with 20 μg/ml proteinase K solution, the sections were labelled with rTdT reaction mix for 1 hour at 37 °C and the reaction was stopped with 2 × SSC. Slides were mounted with DAPI and images were captured using ZEISS LSM 800 confocal microscopy. In order to quantify cell death, the number of TUNEL positive cells in a sample of 500 cells of the outer nuclear layer was counted from five slides (100 cells in each slide from each fish sample) of wildtype and mutant retinas respectively.

### Electron microscopy

Anaesthetised zebra fish (5 days and 13 months old) were fixed in 4% Glutaraldehyde/1% Paraformaldehyde/0.1 M Sodium Cacodylate Buffer for 1.5hrs at room temperature. The specimens were washed twice in 0.1 M Sodium Cacodylate and then post-fixed in 1% Osmium Tetroxide/0.1 M Sodium Cacodylate for 1hr. The Osmium Tetroxide was washed off with three 15mins-changes in distilled water then specimens were stained with 0.5% Uranyl Acetate for 1hr and placed in the dark. The zebrafish were dehydrated through a series of ethanol 30, 50, 70 and 90% for 15 mins each then 100% Ethanol four 10 mins changes and finally dried in 100% Ethanol four 10 mins changes. The specimens were given four 10mins-changes of Propylene Oxide before leaving in a 1:1 PO:EPON resin mix overnight. Over the next day specimens were given 2–3 changes of pure EPON 812 Araldite then freshly embedded in moulds and polymerised at 60 °C for 24–48hrs. Ultrathin sections of 50–60 nm thickness were cut using a Leica UTC Ultratome, then collected on 100 mesh formvar coated copper grids and contrast stained with 2% Uranyl Acetate and Reynolds Lead Citrate for 5 mins each. The sections of zebrafish eyes were viewed on a Tecnai T20 TEM running at 200 kV and DM3/Tiff images captured using Gatan Digital imaging software.

### Electroretinography (ERG)

ERG recording for the zebrafish was carried out according to methods described by Makhankov *et al*.^55^. 7dpf old larvae were dark-adapted for at least 30 minutes before ERG recording. Animals were anaesthetised in tricaine (MS222, Sigma) and immobilized using 0.8 mg/ml Esmeron (Sigma), then placed on top of a piece of wet filter tissue. A straight silver wire was placed beneath the larvae as the reference electrode. To hold the fish down and to provide oxygenation, a small piece of wet tissue was placed on the fish trunk. A glass suction electrode with a ~10 micron tip opening was positioned onto the fish cornea using an MX763OR manipulator from SD Instruments under a Nikon E600FN microscope. ERG recordings were made using Axon Multiclamp 700B amplifier with a gain of 2000, digitized with CED Power 1401 mkII and sampled with Signal 5 software (Cambridge Electronic Design, Cambridge) at 10 kHz. Signals were band-filtered between 3 and 100 Hz. Illumination was provided by a customised battery-powered white LED (50k mcd), controlled using TTL pulses generated through Power 1401 mkII. Test LED illumination was applied after 30 minutes of dark adaption.

### Visual background adaption (VBA) assay

VBA assay was performed as previously described^33^. Briefly, Wildtype and *rpgrip1* ^−/−^ embryos were incubated at 28 °C till 7dpf. Embryos were then put in Petri dishes and underwent a dark adaption for more than 30 min. Then, the Petri dishes were placing on the stand of the microscope and a bright light was shone on the embryos from above. Images were taken immediately when the light was switched on. The light remained on for 15 minutes and again images were taken.

### Western blotting

14dpf wildtype and *rpgrip1* mutant zebrafish heads (with eyes) were homogenised by T-PER^TM^ tissue protein extraction reagent (Thermo Fisher Scientific, UK) with Complete™ protease inhibitor (Roche). Protein concentrations were determined Bio-Rad DC^TM^ protein assay. The equal amounts of total proteins were separated by SDS-PAGE (NuPAGE; 10% gels) which was transferred into nitrocellulose membranes. The membranes were blocked in 5% non-fat milk in TBST at room temperature. Then, the target protein were detected by incubation with primary antibodies including RPGR (1:500, Sigma, HPA001593), GRK1 (1:500, Abcam), GNAT1 (1:500, Gene Tex), Rab8 (1:1000, Sigma) and acetylated tubulin (1:5000, Sigma). After washing, the membranes were incubated with fluorescence IRDye 600–800 RD-conjugated secondary antibodies in TBST. The specific bands were visualized and quantified using Li-cor Odyssey FC and Image Studio software.

### Statistical analysis

All the statistical analysis was carried out using Graphpad Prism (Version 5) (GraphPad Software, www.graphpad.com). An unpaired two tail t test was carried out to determine statistically significant difference between wildtype and *rpgrip1* ^−/−^ groups for *rpgrip1* mRNA analyses and for ERG analyses. For the spatial and temporal gene expression analysis of *rpgrip1* at various developmental stages and different tissues one-way ANOVA with Tukey’s multiple comparison tests was performed using GraphPad prism.

## Electronic supplementary material

Supplementary Figures and Legends
