# Supplementary material for: Rpgrip1 is required for rod outer segment development and ciliary protein trafficking in zebrafish
Source: Sci Rep. 2017 Dec 4;7:16881. doi: 10.1038/s41598-017-12838-x (PMC5715152; doi:10.1038/s41598-017-12838-x)
Supplement: Supplementary file 1 — Supplementary Figures and Legends [file 41598_2017_12838_MOESM1_ESM.pdf]

Rpgrip1 is required for rod outer segment development and ciliary protein trafficking in zebrafish

Rakesh K. Raghupathy[1#], Xun Zhang[1#], Fei Liu[2], Reem H. Alhasani[1], Lincoln Biswas[1], Saeed Akhtar[3], Luyuan Pan[4], Cecilia B. Moens[4], Wenchang Li[5], Mugen Liu[2], Breandan N. Kennedy[6], Xinhua Shu[1,*]

1 Department of Life Sciences, Glasgow Caledonian University, Glasgow G4 0BA, UK

2 Key Laboratory of Molecular Biophysics of Ministry of Education, College of Life Science and Technology, Center for Human Genome Research, Huazhong University of Science and Technology,Wuhan, Hubei 430074, PR China

3 Department of Optometry, King Saud University PO Box 10219, Riyadh 11433, Kingdom of Saudi Arabia

4 Division of Basic Science, Fred Hutchinson Cancer Research Center, Seattle, WA 98109-1024, USA

5 School of Psychology and Neuroscience, University of St Andrews, St Andrews KY16 9AJ, UK

6 UCD Conway Institute & UCD School of Biomolecular and Biomedical Sciences, University College Dublin, Dublin D04 V1W8, Ireland

# These authors contributed equally to this work.

*****Corresponding author**:** Xinhua Shu, Department of Life Sciences, Glasgow Caledonian University, Cowcaddens Road, Glasgow, UK. (e-mail: Xinhua.Shu@gcu.ac.uk)

Supplementary Figures and Legends

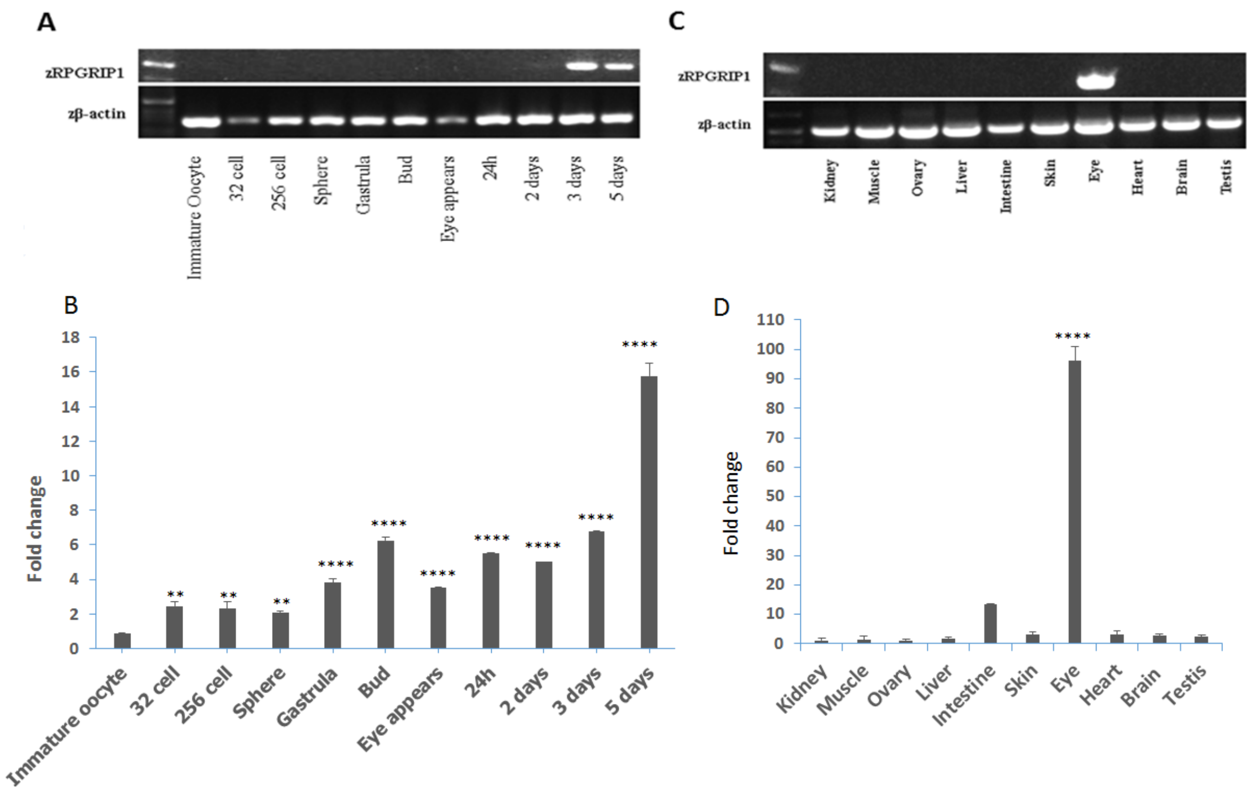

Supplementary Figure S1 Expression of zebrafish *rpgrip11* (*zRPGRIP1*). (A) RT-PCR was used to detect temporal expression of *zRPGRIP1* (upper panel) in different developmental stages. (B) Quantitative gene expression analyses of *zRPGRIP1* in different developmental stages. The fold changes are in comparison to the immature oocyte stage. The result is shown as mean±SD. SD: standard deviation. (C) *zRPGRIP1* expression (upper panel) in different adult tissues detected by RT-PCR. (D) Quantitative gene expression analyses of *zRPGRIP1* in different adult tissues. The fold changes are from comparing to the kidney. ZRPGRIP1 has highest expression in adult eye with about 96 fold. The result is shown as mean±SD. SD: standard deviation.

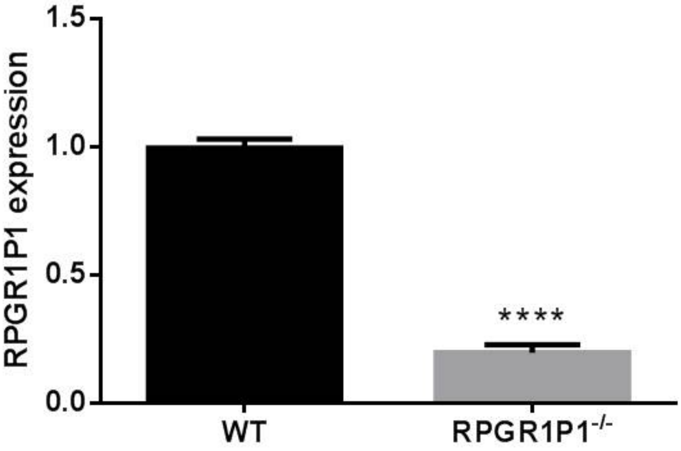

Supplementary Figure S2 *RPGRIP1* expression was significantly reduced in *RPGRIP1*$^{-/-}$mutants, possibly through nonsense mediated mRNA decay. The relative expression of *rpgrip1* in wildtype (WT) and *rpgrip1*$^{-/-}$ mutant zebrafish (5dpf) was measured by quantitative real-time PCR and normalized to β-actin. The result is shown as mean±SD. SD: standard deviation. **** $p<0.0001$.

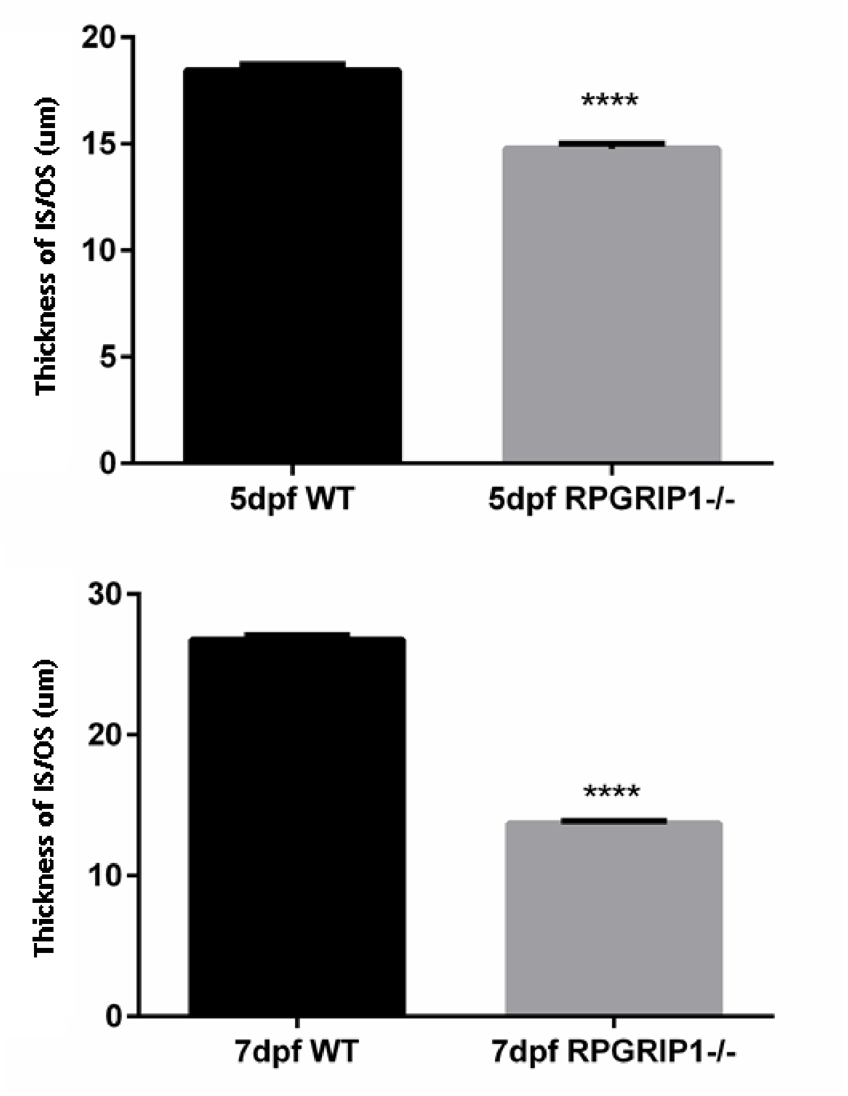

Supplementary Figure S3 The length (in micrometres) of outer/inner segments of central areas of the retinal sections from both wildtype and *rpgrip1*$^{-/-}$ zebrafish at 5 and 7dpf were measured using ZEISS AxioVision software under a light microscopy. The photoreceptor outer segments of *rpgrip1* mutants are significantly shorter, possibly due to the lack of rod outer segment formation. The result is shown as mean±SD. SD: standard deviation. **** p<0.0001.

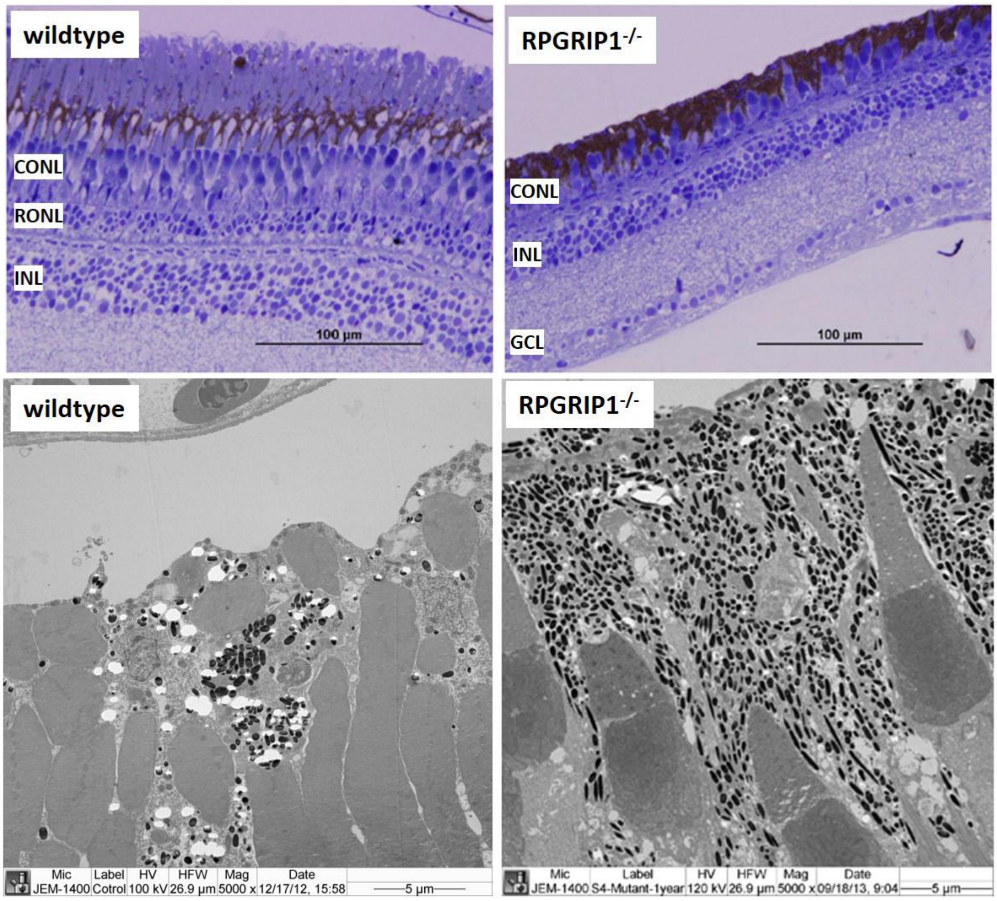

Supplementary Figure S4 Retinal structure of wildtype and *rpgrip1*$^{-/-}$ zebrafish at age of 13 months showed only short cones remained in *rpgrip1*$^{-/-}$ zebrafish retina. Upper panel, light microscopy structure of wildtype and *rpgrip1*$^{-/-}$ zebrafish retinas; lower panel, ultrastructure of wildtype and *rpgrip1*$^{-/-}$ zebrafish retina. CONL, cone outer nuclear layer; GCL, ganglion cell layer; INL, inner nuclear layer; RONL, rod outer nuclear layer.

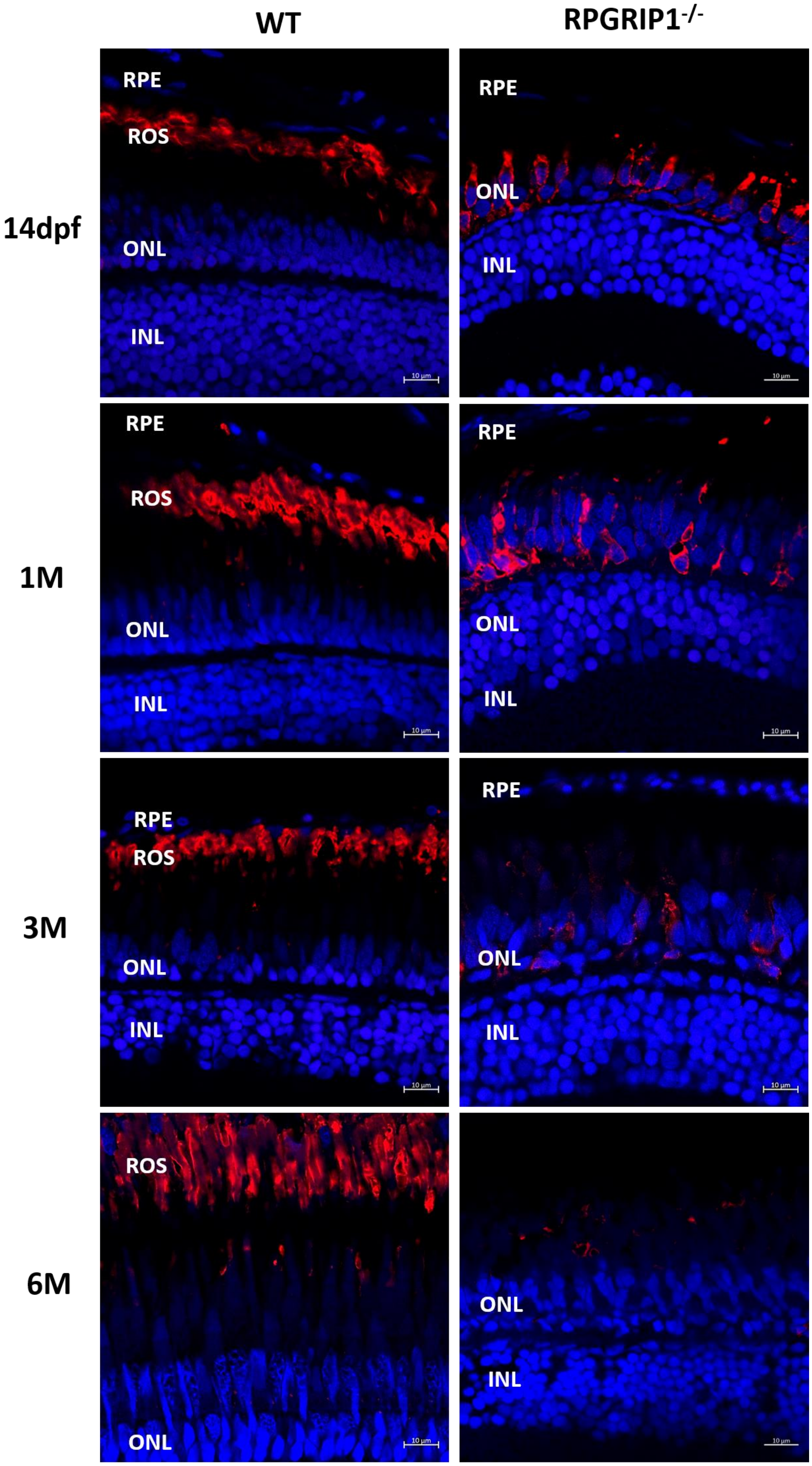

WT
RPGRIP1-/-
14dpf
1M
3M
6M
RPE
ROS
ONL
INL

Supplementary Material, Figure S5 Immunostaining of retinal sections from wildtype and *rpgrip1*$^{-/-}$ zebrafish at age of 14 days (dpf), one month (1 mpf), 3 months (3 mpf) and 6 months (6 mpf). 4D2, labelling rhodopsin, was used for the staining. Nuclei were shown in blue using DAPI. Rhodopsin was mislocalized in all examined *rpgrip1*$^{-/-}$ mutant retina. The signal of rhodopsin was significantly decreased at 1mpf, almost absent at 3mpf, and at background level at 6 mpf. INL, inner nuclear layer; ONL, outer nuclear layer; ROS, rod outer segment; RPE, retinal pigment epithelium.

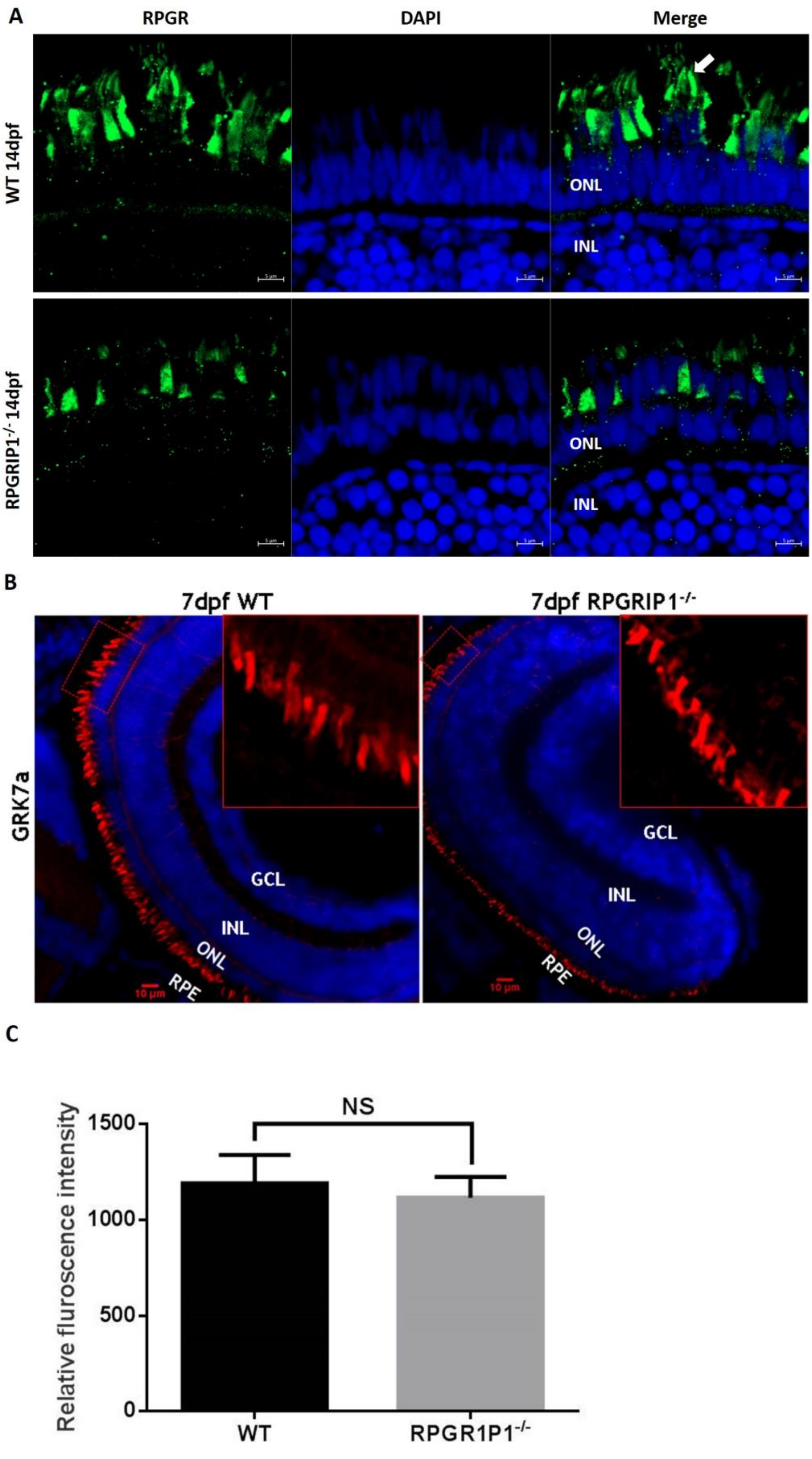
A
RPGR
DAPI
Merge
WT 14dpf
RPGRIP1-/- 14dpf
ONL
INL
B
7dpf WT
7dpf RPGRIP1-/-
GRK7a
GCL
INL
ONL
RPE
10 µm
C
Relative flurosence intensity
NS
1500
1000
500
0
WT
RPGR1P1-/-

Supplementary Figure S6 Immunostaining of retinal sections of wildtype and *rpgrip1*$^{-/-}$ zebrafish using anti-RPGR antibody showed abnormal localization of Rpgr. Rpgr was localized to the connecting cilia and outer segments of photoreceptors in 14dpf wildtype zebrafish retina, while Rpgr was mislocalized in inner segments of photoreceptor cells of *rpgrip1*$^{-/-}$ mutant retinas and the fluorescence signals were significantly decreased. Arrow shows rod outer segment. (B) There is no difference in GRK7a localization to the cone outer segments in both wildtype and *rpgrip1* mutant zebrafish at 5dpf. (C)The fluorescence signals of the boxed areas of wildtype and mutant retinal sections were measured using Image J software. There is no significant difference between wildtyep and mutants with p=0.6989. GCL, ganglion cell layer; INL, inner nuclear layer; ONL, outer nuclear layer; RPE, retinal pigment epithelium. NS, no significance.

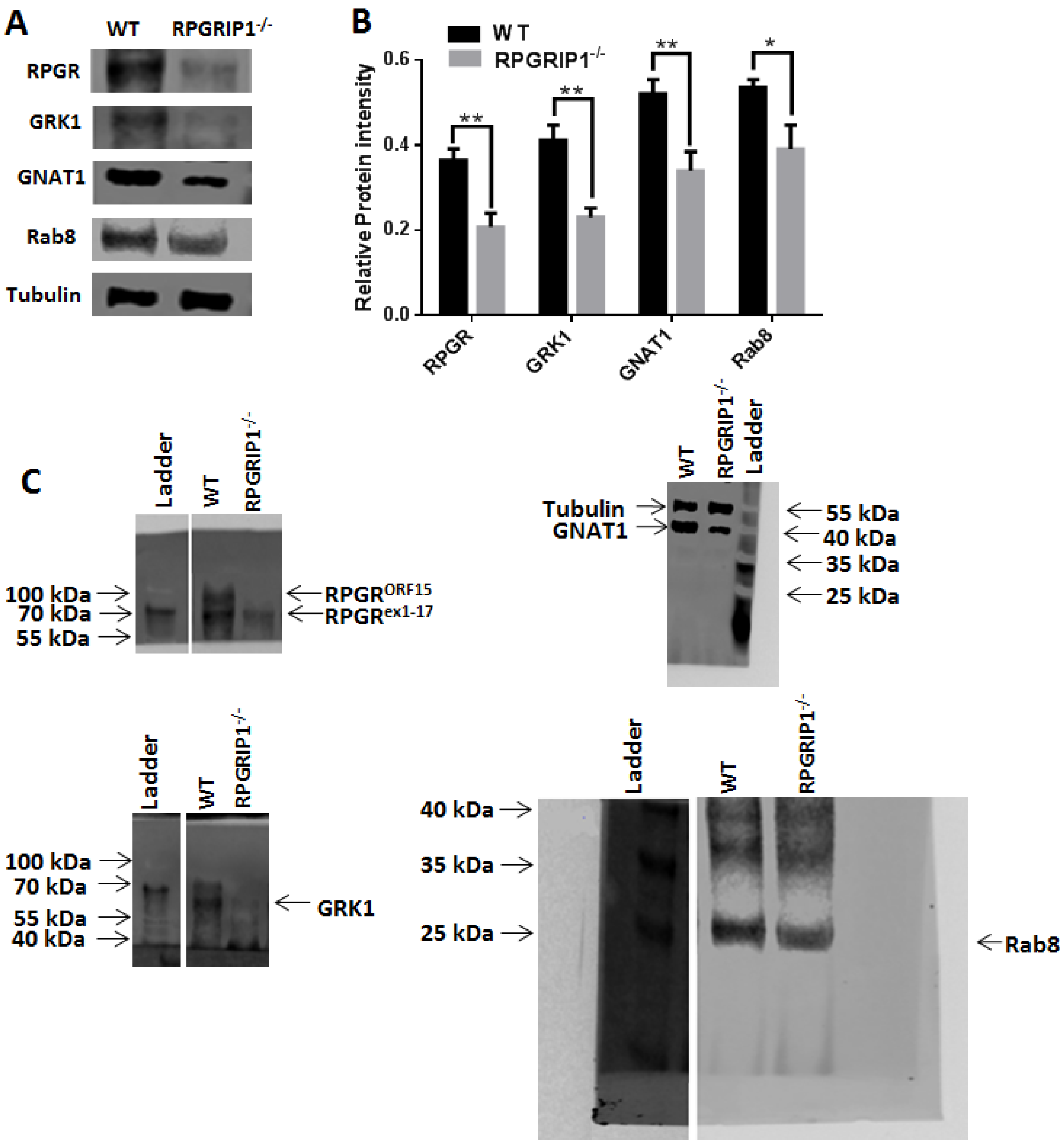

Supplementary Material, Figure S7 Significantly decreased protein level of RPGR, GRK1, GNAT1 and Rab8 in *rpgrip1* mutant zebrafish. (A) The protein of RPGR, GRK1, GNAT1 and Rab8 were detected by western blot in both Wildtype and *rpgrip1* mutant zebrafish where acetylated alpha tubulin was as a house keeping gene. (B) The protein expression of RPGR, GRK1, GNAT1 and Rab8 were normalized to acetylated alpha tubulin and analysed by two ways ANOVA statistical analysis software. Each experiment was performed three times. The data are present as mean±SD. *P<0.05, **P<0.01. (C) Images represent original scans of RPGR, GRK1, GNAT1, Rab8 and Tubulin. RPGRO^{RF15} isoform was disappeared in RPGRIP1$^{-/-}$ mutant zebrafish.
